# An Enzymatically Active β-1,3-Glucanase from Ash Pollen with Allergenic Properties: A Particular Member in the Oleaceae Family

**DOI:** 10.1371/journal.pone.0133066

**Published:** 2015-07-15

**Authors:** María Torres, Oscar Palomares, Joaquín Quiralte, Gabrielle Pauli, Rosalía Rodríguez, Mayte Villalba

**Affiliations:** 1 Biochemistry and Molecular Biology I Department Complutense, University of Madrid, Madrid, Spain; 2 Virgen del Rocío University, Hospital of Seville, Seville, Spain; 3 Hôpital Lyautey, Hopitaux Universitaires de Strasbourg, Strasbourg, France; Henan Agricultural Univerisity, CHINA

## Abstract

Endo-β-1,3-glucanases are widespread enzymes with glycosyl hydrolitic activity involved in carbohydrate remodelling during the germination and pollen tube growth. Although members of this protein family with allergenic activity have been reported, their effective contribution to allergy is little known. In this work, we identified Fra e 9 as a novel allergenic β-1,3-glucanase from ash pollen. We produced the catalytic and carbohydrate-binding domains as two independent recombinant proteins and characterized them from structural, biochemical and immunological point of view in comparison to their counterparts from olive pollen. We showed that despite having significant differences in biochemical activity Fra e 9 and Ole e 9 display similar IgE-binding capacity, suggesting that β-1,3-glucanases represent an heterogeneous family that could display intrinsic allergenic capacity. Specific cDNA encoding Fra e 9 was cloned and sequenced. The full-length cDNA encoded a polypeptide chain of 461 amino acids containing a signal peptide of 29 residues, leading to a mature protein of 47760.2 Da and a pI of 8.66. An N-terminal catalytic domain and a C-terminal carbohydrate-binding module are the components of this enzyme. Despite the phylogenetic proximity to the olive pollen β-1,3-glucanase, Ole e 9, there is only a 39% identity between both sequences. The N- and C-terminal domains have been produced as independent recombinant proteins in *Escherichia coli* and *Pichia pastoris*, respectively. Although a low or null enzymatic activity has been associated to long β-1,3-glucanases, the recombinant N-terminal domain has 200-fold higher hydrolytic activity on laminarin than reported for Ole e 9. The C-terminal domain of Fra e 9, a cysteine-rich compact structure, is able to bind laminarin. Both molecules retain comparable IgE-binding capacity when assayed with allergic sera. In summary, the structural and functional comparison between these two closely phylogenetic related enzymes provides novel insights into the complexity of β-1,3-glucanases, representing a heterogeneous protein family with intrinsic allergenic capacity.

## Introduction

Beta-1,3-glucanases [glucan endo-1,3-β-D-glucosidases (EC 3.2.1.39)] constitute a ubiquitous family of proteins widely distributed among higher plants, fungi and bacteria. The biochemical activity of these enzymes is based on the hydrolytic cleavage of 1,3-D-glucosidic linkages between β-1,3-glucans, which are major components of the cell wall surrounding fungi and plants. The enzymatic activity of β-1,3-glucanases is crucial in the chemical changes of the glucan composition and in the remodeling of the cell wall. In fungi, these enzymes play a role in cell expansion, cell-cell fusion and spore release [1]; in bacteria, assimilating fungal cell wall as food [2] and developing metabolic functions [3], and in plants, participating in specialized stages of their development such as cell division [4], microsporogenesis [5], pollen tube growth or seed germination [6].

Beta-1,3-glucanases from higher plants belong to the glycosyl hydrolase family 17 (GHF 17) [7, 8]. They exist in multiple forms differing on their molecular properties (molecular mass, isoelectric point, primary structure, and glycosylation) [9], cellular localization and expression pattern, supporting the diverse roles for these enzymes in plants[10]. Protective responses against pathogen attack [6, 11, 12] have been attributed to the expression of these enzymes that belong to the group 2 of pathogenesis-related (PR) proteins [11, 12]. Their antifungal activity in plants has made these enzymes important tools for strategies to develop resistance in crop plants against fungal pathogens [13]. Beta-1,3-glucanases are also constitutively expressed in several tissues of plants and have been implicated in physiological and developmental processes in organs such as bud, style of flower, anther, seed from uninfected plants, *e*.*g*. cell division or pollen development.

An allergenic character has been associated to several members of this protein family, either from pollens [14], fruits [15] or natural rubber latex [16]. The crucial roles of these molecules acting as allergens and triggering the symptoms among allergic patients make their characterization relevant for the diagnosis of such hypersensitivity disorder. Pollens constitute main sources of aeroallergens involved in Type I allergies, and those induced by *Oleaceae* affect many European countries, being olive tree the predominant species in the Mediterranean area and ash in zones of North and Center Europe [17, 18]. The relevance of Ole e 9-the olive β-1,3-glucanase- depends on the pollen gradient, reaching a recognition frequency higher than 65% among the olive pollen allergic population living in regions of high pollen density[14, 19]. Their implications in pollen-plant food-latex IgE cross-reactivity processes add a clinical relevance to this enzyme [20, 21].

Considering the length of their polypeptide chain, short (33 to 41 kDa) and large (45 to 50 kDa) β-1,3-glucanases have been reported. Whereas the former are constituted exclusively by the catalytic domain, the latter present an additional carbohydrate-binding module (CBM) that is placed at the C-terminus. The enzymatic activity of long glucanases seems to be significantly lower than that of short ones, which are generally basic proteins [22]. CBMs are grouped into 55 families that show notable variation in ligand specificity because their functions are miscellaneous [23].

Ole e 9 is a long β-1,3-glucanase of 46 kDa found in pollen grains [14], composed of one glycosylated polypeptide chain with two independent domains (36 kDa and 10 kDa). The N-terminal domain (NtD), which was produced in *Pichia pastoris* yeast [20], contains the active site and a 3D-framework highly conserved in these enzymes. The C-terminal domain (CtD), which was also produced in *P*. *Pastoris* [24] and its 3D-structure determined [25], could participate in the catalysis as a regulatory module [26]. It belongs to CBM-43 family as its homologue Ole e 10 [27], an allergen from olive pollen. Both recombinant domains retain the allergenic character of the whole allergen [28].

In this work, we report the cloning and sequencing of an allergenic β-1,3-glucanase from ash pollen. This allergen adds a new member to the diagnosis panel from ash pollen. The recombinant production of both catalytic and regulatory domains has been included together with their purification, structural and functional characteristics. Their particular catalytic features are compared with those of its olive pollen counterpart, Ole e 9. Despite the notable differences between them, both conserve their allergenic properties.

## Materials and Methods

### Biological materials

Sera from olive pollen-allergic patients from Jaén (Spain) sensitized to Ole e 9 as well as ash-pollen allergic patients from Strasbourg (France) were employed for the immunological characterization of recombinant NtD of Fra e 9 (rNtD-Fra e 9) and CtD of Fra e 9 (rCtD-Fra e 9). Polyclonal antibodies (pAb) against rNtD and rCtD from Ole e 9 and Fra e 9 were obtained as described [29]. The clinical investigation has been conducted according to the principles expressed in the Declaration of Helsinki. Written informed consent has been obtained from the patients. The Ethical Committee of the Complutense University (Madrid, Spain) has approved the protocols used for this experimental work and all the methodology related to the use of human sera in this study.

Pollen was purchased from Allergon-Pharmacia (Ängelholm, Sweden). Pollen protein extracts were obtained by saline extraction as described previously [30]. Protein extract concentrations were determined by Lowry method as described [31].

### Bacterial strains and plasmids

The TOPO TA-cloning kit (Invitrogen, Groningen, The Netherlands) was used for cloning the PCR products. *P*. *pastoris* competent cells KM71 (Invitrogen) and *E*. *coli* competent cells BL21Gold(DE3) (Stratagene, La Jolla, CA, USA) were used for expression of rCtD-Fra e 9 and rNtD-Fra e 9, respectively.

### Cloning of the β-1,3-glucanase from ash pollen and their independent domains

Total RNA was extracted from ash pollen and cDNA was synthesized with the SMART RACE cDNA amplification kit (BD Biosciences-Clontech, Madrid, Spain). Fra e 9-specific DNA sequence was obtained by several PCR rounds with the primers listed in S1 File. After the first PCR round with degenerate primers Fra9.1 and Fra9.2, an internal fragment of 717 nucleotides was obtained. Fra9.3 was designed based on this sequence to amplify the rest of the 5’ and 3’coding regions together with the nonspecific primer UPM, included in the cDNA amplification kit. Finally, the DNA fragment encoding the mature protein was amplified in a last PCR round using the sense Fra9.4 and the antisense CtD.2 primers.

Both domains of this β-1,3-glucanase were cloned in order to get them independently expressed in the appropriate heterologous expression systems. The DNA encoding rNtD-Fra e 9 (residues 30–349) was amplified by PCR using NtD.1 and NtD.2 primers. The sense primer includes an *Nde*I restriction site and the antisense primer sequence, six histidine residues to purify the protein, followed by a stop codon and an *EcoR*I restriction site. The DNA fragment was directly cloned in the pCR2.1 vector included in the TOPO TA-cloning kit, and used to transform TOP10 *E*. *coli* competent cells. The pCR2.1/NtD recombinant construct was digested with *Nde*I/*EcoR*I restriction enzymes, and subcloned in the expression vector pET11b.

For amplification of the DNA encoding the C-terminal domain of Fra e 9, comprising amino acids 350–461, primers CtD.1 and CtD.2 were used. The forward primer includes an *Xho*I restriction site and the reverse primer a stop codon and a *Not*I restriction site. Recombinant construct was digested with *Xho*I/*Not*I restriction enzymes and the DNA fragment was subcloned into the same sites of the plasmid pPICZαA.

### Overexpression and purification of rNtD-Fra e 9 and rCtD-Fra e 9

*E*. *coli* strain BL21Gold(DE3)-competent cells were transformed with pET11b/NtD and then cultured overnight at 37°C in LB/Amp medium. Cultures were tempered at 25°C and induced overnight with 0.4 mM IPTG. The cellular pellet was resuspended in 50 mM Tris-HCl, pH 7.4 containing 1 mM PMSF, and then disrupted by sonication and centrifuged. The soluble fraction was dialyzed and then applied onto a Ni^2+^-chelating affinity column (HisTrap FF crude column, GE Healthcare Bio-Sciences AB, Uppsala, Sweden) and the protein was eluted using a 20 mM to 0.5 M imidazole gradient. Selected fractions were subjected to a cation exchange chromatography in a HiTrap CM FF column (GE Healthcare) and being eluted with a gradient from 50 mM to 1 M sodium acetate pH 5.0.

The pPICZαA/CtD construct was linearized with *BstX*I restriction enzyme, and transformed into *P*. *pastoris* strain KM71. Recombinant clones were selected in YPDS plates with 100 μg/ml zeocin. Transformed strains were cultured for 72 h at 30°C in buffered glycerol complex medium, and then processed as reported previously [24, 29]. The extracellular medium after induction was dialyzed against 20 mM ammonium bicarbonate, pH 8.0, and lyophilized. Two chromatographies, a Sephadex G-50 column in 0.2 M ammonium bicarbonate and a DEAE-cellulose using a gradient of 20 mM to 0.5 M of ammonium bicarbonate were used for the protein purification.

### MS analysis and spectroscopic characterization

Molecular masses were determined by a MALDI-TOF performed in a Bruker-Reflex IV MALDI-TOF (Bruker-Franzer Analytic, Bremen, Germany). Far-UV CD spectra were recorded at 20°C in a Jasco J-715 spectropolarimeter (Japan Spectroscopic Co., Tokyo, Japan) as previously described [32] with minor modifications. Proteins were prepared at 0.2 mg/ml in 50 mM sodium phosphate pH 7.0. The spectra were measured in a cylindrical cell with a 0.1cm optical path length. Mean residue mass ellipticities were calculated based on 105.2 for rNtD and 107.9 for rCtD as the average molecular mass/residue and expressed in terms of mean residue molecular weight ellipticity, [θ]_MWR_ (degree x cm^2^ x dmol^-1^). Thermal unfolding of both proteins was measured by registering the molar ellipticity at 220 nm while heating 1°C/min from 20°C to 80°C.

### Electrophoresis, Immunoblotting and ELISA

SDS-PAGE was performed in 15% or 17% polyacrylamide gels under non-reducing conditions. Purified proteins and extracts were visualized by Coomassie Brilliant Blue staining or transferred to nitrocellulose membranes (Hybond ECL, GE Healthcare) to be immunostained with specific pAbs followed by a horseradish peroxidase (HRP)-labeled goat anti-rabbit IgG (diluted 1:2500, Bio-Rad, Richmond, CA, USA). Sera from olive pollen-allergic and ash pollen-allergic patients (diluted 1:10) with positive reaction to Ole e 9 were also used. IgE binding was detected with the mouse anti-human IgE antibody (diluted 1:5000, kindly donated by ALK-Abelló, Madrid, Spain), followed by a HRP-labeled rabbit anti-mouse IgG (1:2500, Dako, Copenhagen, Denmark). Peroxidase reaction was developed with ECL Western blotting substrate (Pierce Chemical Co, Rockford, IL, USA). Concanavalin A (ConA) staining was performed as described previously [33].

ELISA inhibition assays were performed in microtitre plates coated with 0.2 μg/well of rNtD or 0.1 μg/well rCtD and sera from ash-allergic patients, as described [34] and increasing amounts of ash or olive pollen extracts up to 250 μg. Peroxidase reaction was developed with the substrate OPD (*o*-phenylenediamine dihydrochloride) and optical density (OD) was measured at 490 nm. Each value was the mean of two determinations after blank subtraction. Percentage of IgE inhibition was calculated considering the IgE-binding signal obtained in the absence of inhibitors as 100%.

Two-dimensional electrophoresis (2-DE) [33] was performed with 300 μg of olive or ash pollen extracts using for the first dimension IPG strips (7 cm) containing an immobilized linear pH gradient from 3 to 10 (Bio-Rad Laboratories, Hércules, CA, USA) according to the manufacturer’s protocol. The second dimension in SDS-PAGE was performed in the presence of the reducing agent dithiothreitol (DTT).

### Enzyme activity assay

Beta-1,3-glucanase activity and kinetic parameters of the rNtD were determined as described [14] measuring the glucose-reducing equivalents at 540 nm released from the β-1,3-glucan laminarin from *Laminaria digitata* or the β-1,3/1,4-glucan lichenan (2 mg/ml) (Sigma-Aldrich) after the incubation with both rNtD-Ole e 9 and rNtD-Fra e 9 (5 μg) using laminarinase as a standard. The reaction was performed in 50 mM sodium acetate, pH 5.0, containing 400 μg/ml of BSA during 1 h at 37°C. The enzymatic reaction was stopped by the Somogyi alkaline copper tartrate reagent [35] heating the samples in a boiling water bath for 15 min. The color developing was obtained by means of the Nelson reagent [36]. One enzymatic unit was defined as the amount of enzyme required to release 1 μmol of reduced groups per minute under the standard assay conditions. The pH dependence of enzyme activity was measured under the same conditions from pH 3.5 to 8.0 at intervals of 0.5 units.

### Carbohydrate-binding assay

The polysaccharide-binding activity of the rCtD from Fra e 9 and Ole e 9 was tested by affinity gel electrophoresis (AGE) as described previously [27]. Proteins (2 μg) were electrophoresed in native 15% polyacrylamide gels containing laminarin. Gels without ligand were electrophoresed simultaneously. BSA (0.7 μg) was used as a negative control.

### Alignments and 3D modeling

Multiple amino acid sequence alignments were performed with CLUSTAL-W and displayed with GeneDoc. Molecular modeling was carried out with PyMol (W.L. DeLano (http://pymol.org)).

## Results

### Detection of a β-1,3-glucanase in ash pollen

**Fig 1:**
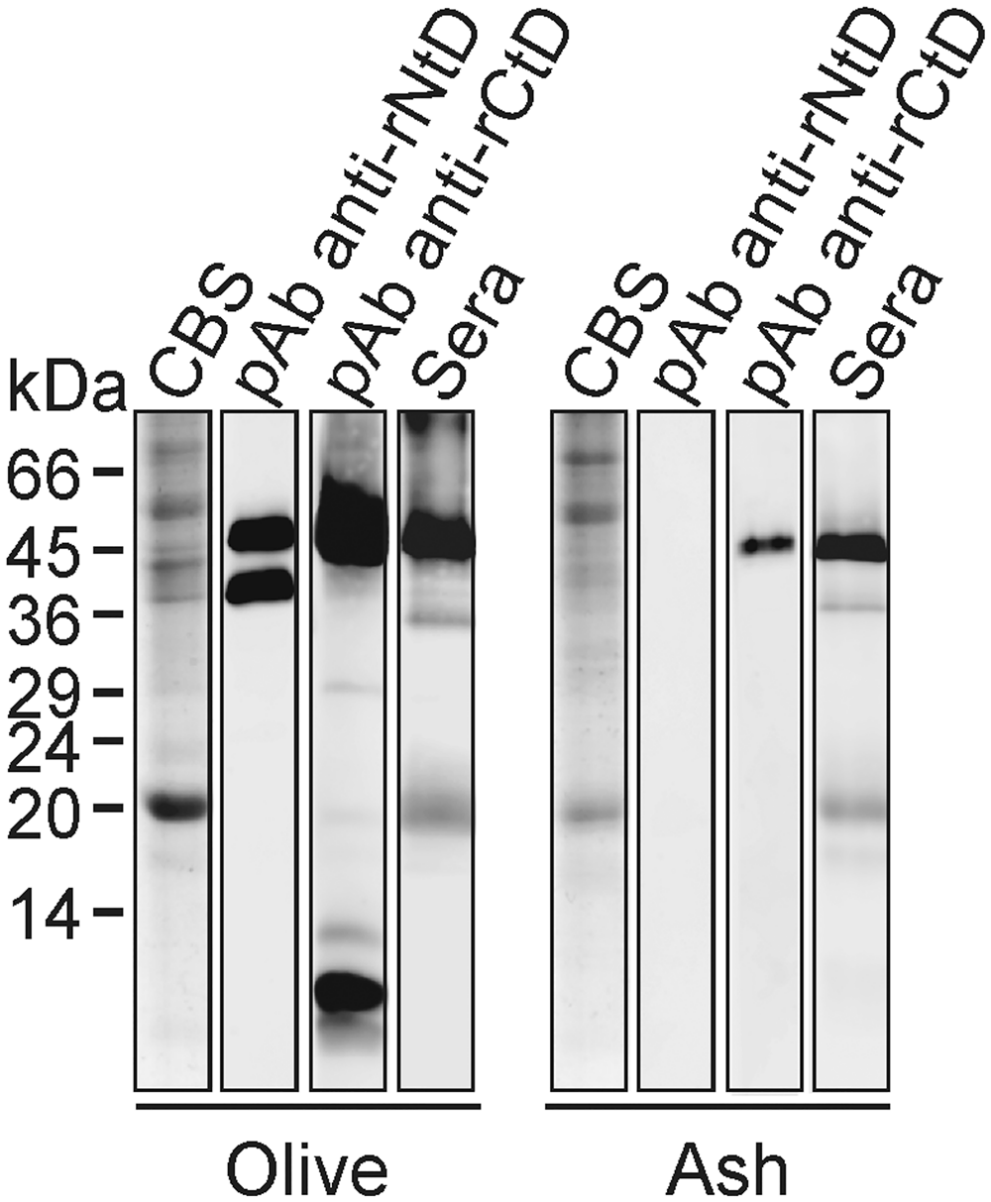
Detection of a β-1,3-glucanase homolog to Ole e 9 in ash pollen. Coomassie Blue staining (CBS), IgG reactivity of pAbs obtained agaisnt rNtD and rCtD of Ole e 9 and IgE reactivity of a pool of sera from patients allergic to olive pollen, to olive and ash pollen extracts (40 μg of total protein). Molecular mass markers are indicated.

Specific pAbs against the catalytic and carbohydrate-binding domains of Ole e 9, the β-1,3-glucanase from olive pollen, demonstrated the existence of homologous counterparts in ash pollen by immunoblotting (Fig 1). The rNtD-specific pAb did not recognize any band in the ash pollen extract in the assayed conditions. However, the rCtD-specific pAb recognizes bands around 45 kDa, the molecular mass expected for these enzymes and a band over 10 kDa. The latter probably corresponds to an ash homologue of Ole e 10, a carbohydrate-binding protein with high sequence identity to the CtD of Ole e 9. To analyse the IgE-binding capacity of this family of enzymes, IgE binding by Western blot with a pool of sera from patients allergic to Ole e 9 was tested (Fig 1) and the results suggested the existence of an allergenic glucanase in ash pollen.

### Molecular cloning and sequencing of the ash pollen β-1,3-glucanase

**Fig 2:**
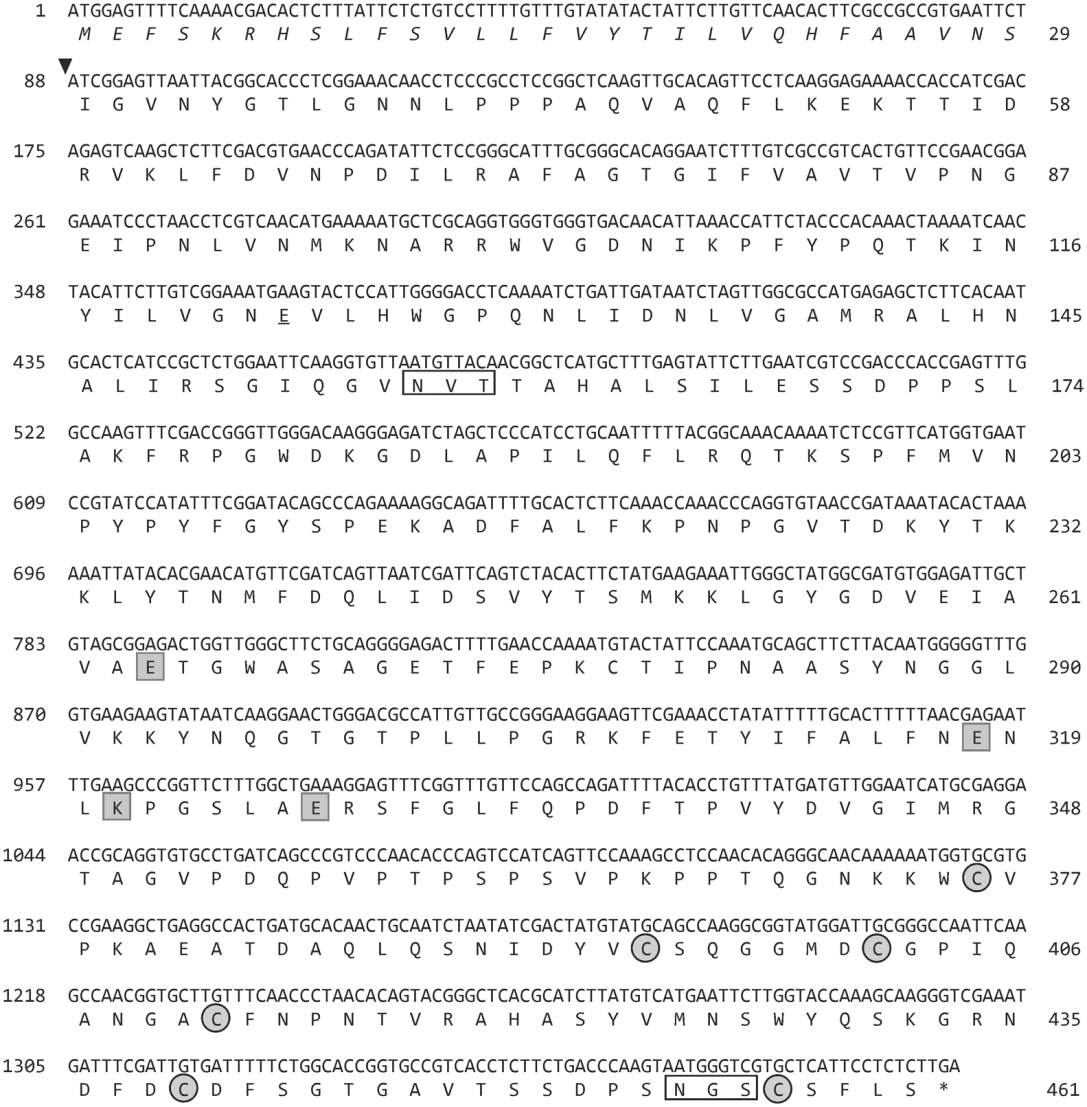
Nucleotide sequence of cDNA encoding Fra e 9 and deduced amino acid sequence. The putative cleavage site for the signal peptide is indicated by an arrowhead. The catalytic residues are boxed. The cysteine residues of the C-terminal domain are circled. The potential N-glycosylation sites are framed.

Total cDNA from ash pollen was used as a template for PCR amplification of the Fra e 9 codifying sequence. The full-length cDNA of 1383 bp encoded a 461 amino acid sequence (Fig 2), which N-terminus contains a signal peptide sequence. A predicted cleavage site for processing after the 29^th^ amino acid (Ser), leads to a mature protein of 47760.2 Da with a calculated pI of 8.66. The Fra e 9 sequence includes two potential N-glycosylation consensus sites (NXS/T), -Asn-156 at the NtD, and Asn-454 at the CtD-. The conserved amino acid tetrad (Glu-235, Glu-289, Lys-292 and Glu-298) of the catalytic site, as well as the characteristic six cysteine pattern of the C-terminal module were present in the structure.

**Fig 3:**
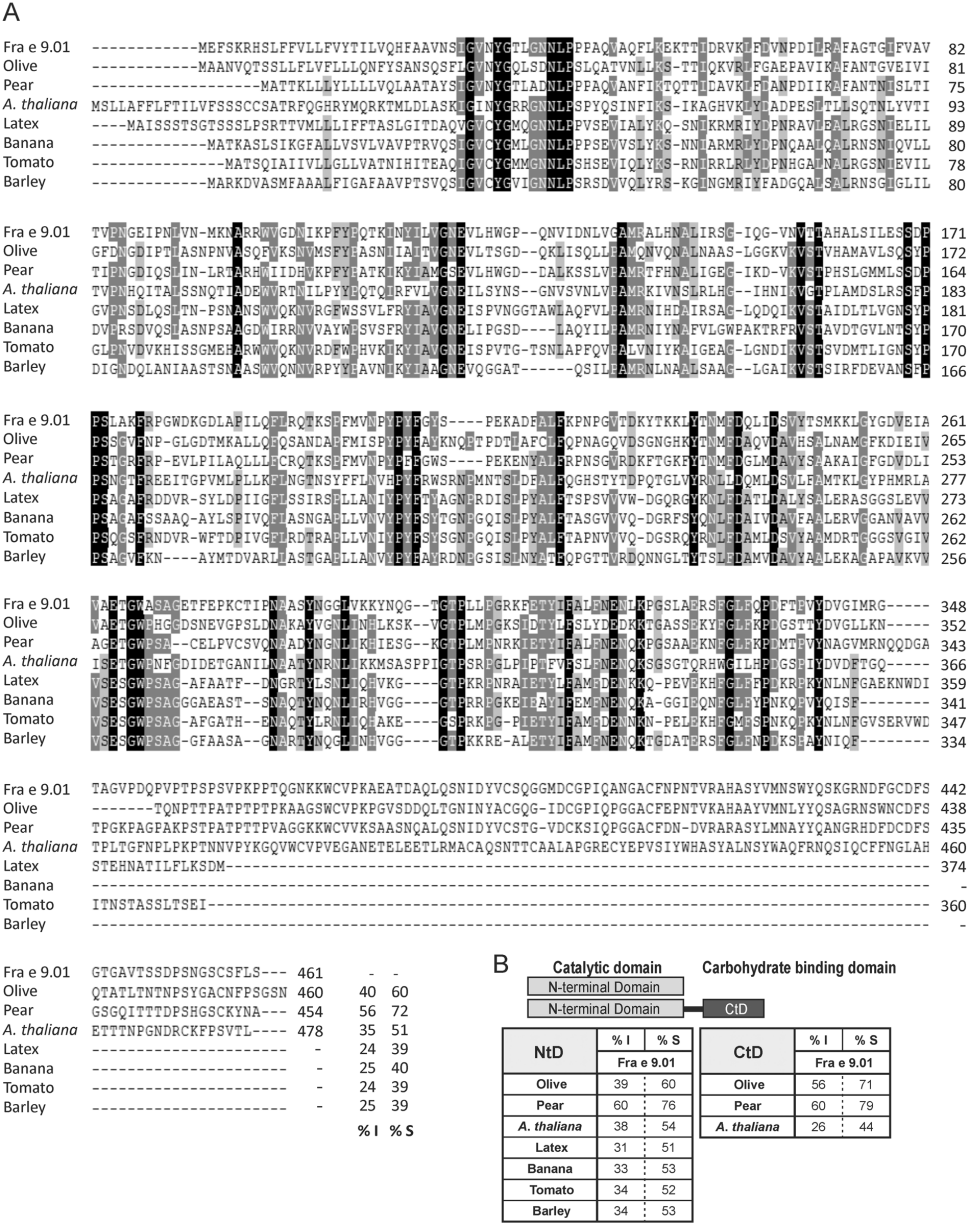
Alignment of Fra e 9 with the β-1,3-glucanases from other plant sources. Ash (Fra e 9, KC920916), olive (Ole e 9, Q94G86), pear (B9VQ36), *A*. *thaliana* (Q06915), latex (Hev b 2, A2TJX4), banana (Mus a 5, A7U7Q7), tomato (Q01413), barley (P15737). (A) Dashes indicate gaps. Letters over black shading are conserved residues in all sequences; dark gray indicates residues conserved in at least six sequences; light gray indicates residues conserved in five sequences. % I and %S represent identity and similarity percentages of these sequences comparing to that of Fra e 9. (B) Independent comparison of the amino acid sequences of N-terminal and C-terminal domains of the β-1,3-glucanases with Fra e 9 domains.

The amino acid sequence alignment with β-1,3-glucanases available in the GenBank database (Fig 3A) showed highest identity with that of pear (56%), being only a 40% the identity with Ole e 9, and 35% with A6, a long β-1,3-glucanase described in *Arabidopsis* anthers. Lower identity degree is obtained (Fig 3B) when the NtD is itself compared either with the same enzymes (39% olive and 38% *Arabidopsis*) or with other short glucanases such as those of latex (31%) and banana (33%) both described as allergens, or tomato (34%) and barley (25%). The identity degree between the CtD of Ole e 9 (360–460) and Fra e 9 (354–461) is of 56% (Fig 3B).

### Recombinant expression of rNtD-Fra e 9 and rCtD-Fra e 9, purification and characterization

**Fig 4:**
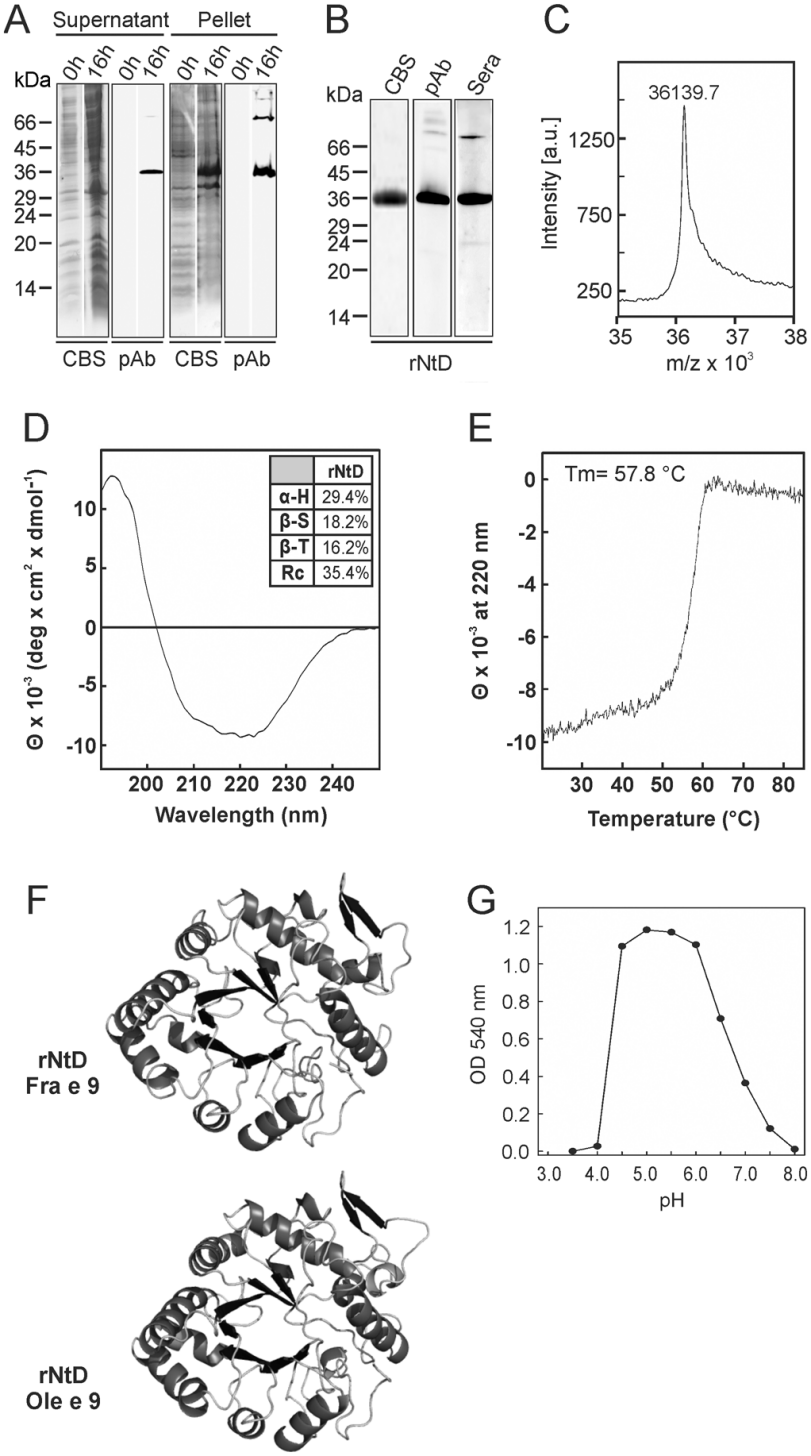
Recombinant expression and molecular and functional characterization of the NtD-Fra e 9. (A) Time-course of the expression of rNtD-Fra e 9 in *E*. *coli*. Supernatants and pellets from cultures were harvested at different times after induction and stained with Coomassie Blue (CBS) after SDS-PAGE. (B) Purified domain was analysed by CBS and by immunostaining with a pool of sera from olive pollen allergic patients (Sera) or specific antiserum (pAb). (C) Mass spectrometry analysis. (D) CD spectrum in the far-UV (190–250 nm). In the inset are included the porcentages of secondary structure. (E) Thermal unfolding measured as ellipticity at 220nm during heating from 20°C to 80°C. (F) Schematic representation of the 3D-model of NtD-Fra e 9 and NtD-Ole e 9; The main structural differences are highlighted. (G) The pH dependence for the enzymatic activity of rNtD-Fra e 9 was assayed at different pH values.

The NtD (residues 30 to 349) and CtD (residues 350 to 461) of Fra e 9 were independently produced as recombinant proteins in *E*. *coli* bacteria and *P*. *pastoris* yeast, respectively. Although rNtD-Fra e 9 was obtained mainly in inclusion bodies (Fig 4A), the protein of around 36 kDa after 16 h IPTG induction at 25°C was also present in the soluble fraction, which was employed as starting material for purification of the rNtD-Fra e 9. The protein purified by two affinity and cation exchange chromatographic steps displayed IgG- and IgE-binding capacity when using specific pAb and sera from allergic patients sensitive to β-1,3-glucanase (Fig 4B). MS analysis yielded a main peak of 36139.7 Da (Fig 4C), which fits well with the predicted molecular mass of 36114.3 Da based on the amino acid composition. CD spectra in the far-UV range are shown in Fig 4D. The secondary structure content for rNtD rendered 29.4% α-helix, 18.2% β-sheet, 16.2% β-turn and 35.4% random coil. The temperature-dependent unfolding was registered at 220 nm, and the obtained melting temperature (Tm) was of 57.8°C (Fig 4E). In fact, after 1 h at 50°C a 50% of the activity is lost. Fig 4F shows the 3D-modeling of the NtD-Fra e 9 built based on the banana β-1,3-glucanase [37], showing the conformation of a (β/α)_8_TIM-barrel motif characteristic of this family of enzymes. A superposition of both polypeptide chains shows nearly identical secondary structures. Recombinant NtD-Fra e 9 exhibited β-1,3-glucanase activity as it was able to hydrolyze the β-1,3-glucan laminarin. A suitable amount of 138 pmoles was determined for rNtD-Fra e 9 in the presence of laminarin as a substrate. The activity of rNtD was maintained between pH 4.5 and 6.0 (Fig 4G), showing the maximal specific activity of 959.4 μg of glucose released/min/μmol of protein at pH 5.0. rNtD-Fra e 9 did not show β-1,3-glucanase activity when lichenan, a β-1,3/1,4-glucan, was used as a substrate (data not shown).

**Fig 5:**
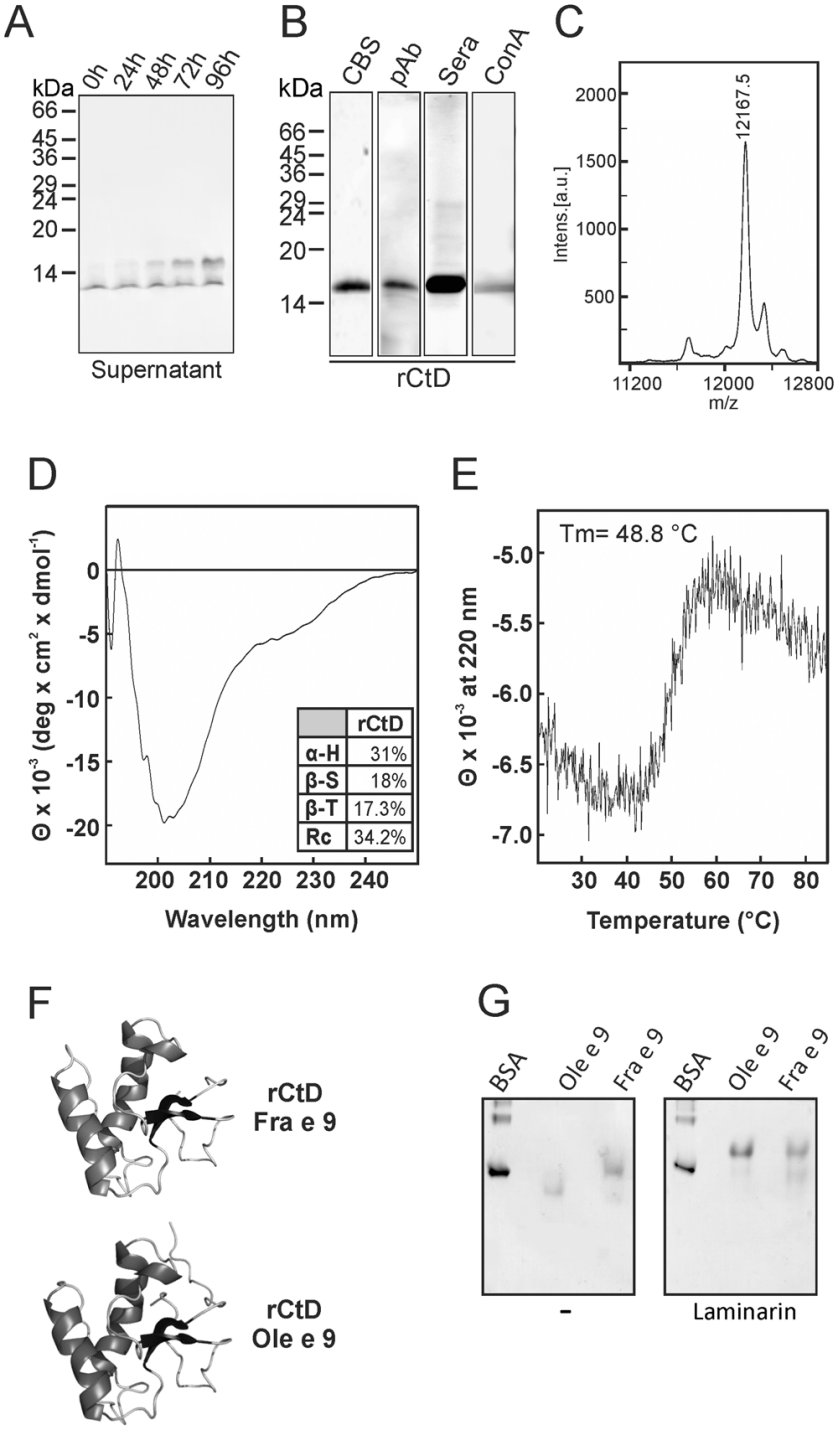
Recombinant expression and molecular characterization of CtD-Fra e 9. (A) Time-course of the expression of rCtD-Fra e 9 in *P*. *pastoris*. Extracellular medium was harvested at different times after induction and stained with Coomassie Blue (CBS) after SDS-PAGE. (B) Purified domain was analysed by CBS, immunostaining with a pool of sera from olive pollen allergic patients (Sera) and with a Ole e 9-specific antiserum (pAb), and staining with ConA-rNtD was loaded in the same lane of the gel as a control for no glycosylation-. (C) Mass spectrometry analysis. (D) CD spectra in the far-UV (190–250 nm). (E) thermal unfolding assay during heating from 20°C to 80°C as in Fig 4. (F) Schematic representation of the 3D of Fra e 9 modeled against Ole e 9 3D structure determined by NMR [26]. (G) Affinity gel electrophoresis (AGE) analysis of rCtD-Fra e 9 (Fra e 9), rCtD-Ole e 9 (Ole e 9) and BSA as negative control; both proteins were electrophoresed under non-denaturing conditions in polyacrylamide gels in the presence and absence (-) of laminarin.

The rCtD-Fra e 9 was obtained as a secretion protein in the extracellular medium of *P*. *pastoris* where a band of around 15 kDa was detected after 48 h with a maximal level at 96 h of induction (Fig 5A). The band appeared below the 15 kDa corresponded to the migration front of the gel. After gel filtration chromatography and RP-HPLC, the purified rCtD-Fra e 9 was able to bind IgG and IgE with specific pAbs against rCtD-Ole e 9 and sera of patients sensitive to β-1,3-glucanases (Fig 5B). Glycosylation of rCtD-Fra e 9 was confirmed by ConA staining (Fig 5B). A heterogeneous profile obtained by MS displayed several peaks (Fig 5C). The major peak corresponds to a protein exhibiting 12167.5 Da, which compared to the 11364.4 Da of the clone-deduced sequence, could indicate the presence of a small oligosaccharide structure (803.1 Da) attached to the single N-glycosylation site at Asn-454. The rCtD-Fra e 9 contains 31% α-helix, 18% β-sheet, 17.3% β-turn and 34.2% random coil (Fig 5D) and a Tm of 48.8°C (Fig 5E). The modeling was made using the coordinates of the rCtD-Ole e 9 structure, determined by NMR (Fig 5F) [25]. It would consist of two 〈-helices and a small antiparallel β-sheet that are connected by several loop segments.

The ability of rCtD-Fra e 9 to bind the soluble polysaccharide laminarin at a concentration of 2.5 mg/ml was assessed by quantitative AGE under non-denaturing conditions. Purified rCtD-Fra e 9 displayed a significant but lower binding capacity specifically to laminarin (Fig 5G) than rCtD-Ole e 9, as demonstrated by the clear shift of mobility in AGE when laminarin is embedded in the gel.

### IgG and IgE epitopes of rFra e 9 are present in their natural counterparts from olive and ash pollen extracts

**Fig 6:**
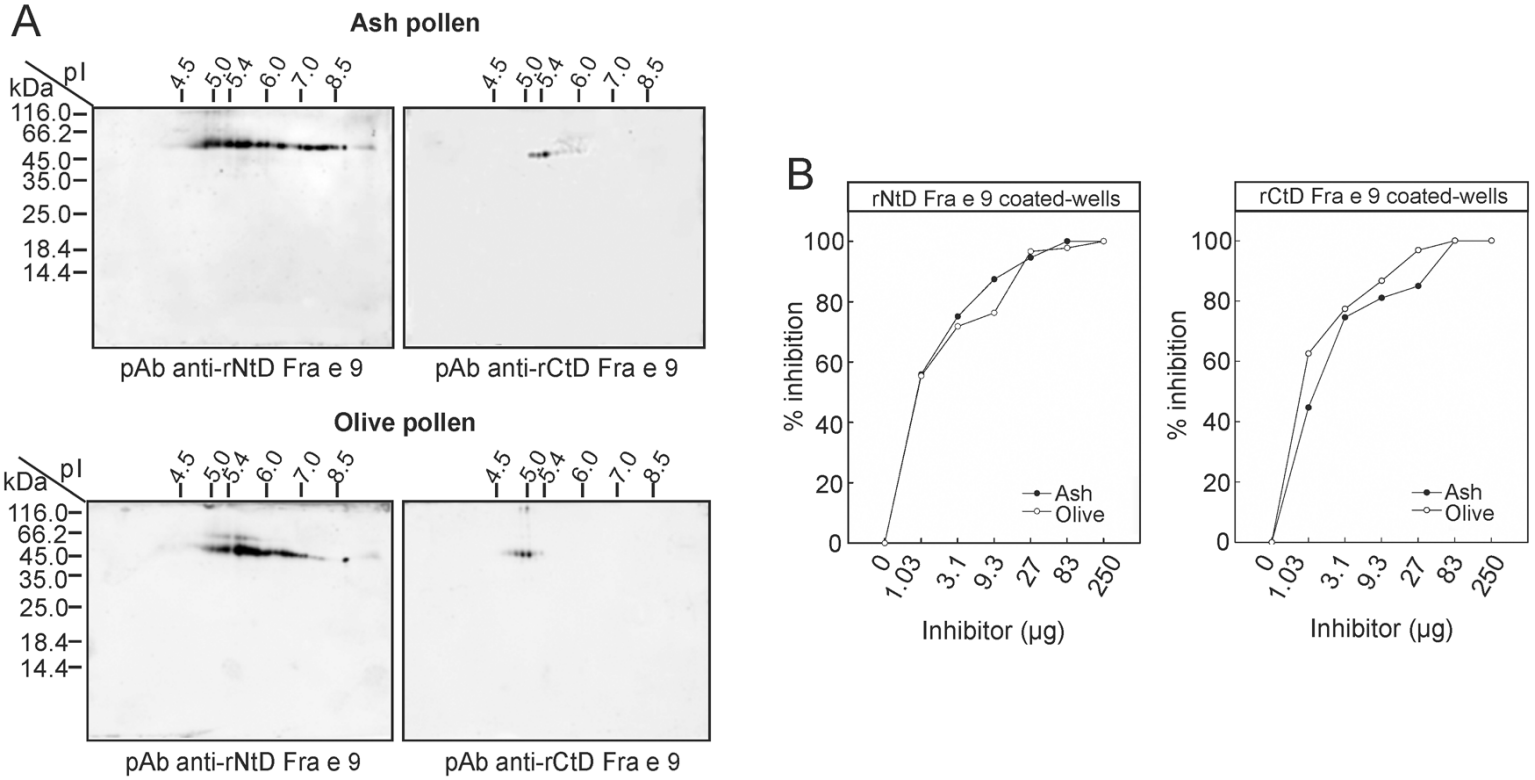
Analysis of the IgG and IgE reactivity of Fra e 9 and Ole e 9. (A) Identification of the isoforms pattern of both β-1,3-glucanases by 2-DE with pAbs raised against rNtD and rCtD of Fra e 9 in ash and olive pollen extracts. (B) IgE-binding inhibition analysis by ELISA of Fra e 9 recombinant domains after preincubation of the pool of sera from patients allergic to olive with increasing amounts of ash or olive pollen extracts.

Specific rabbit antisera raised against rNtD and rCtD of Fra e 9 were used to analyse the correlation with the natural forms in ash and olive pollen extracts by2-DE (Fig 6A). Although several isoforms with pI ranging between 4.8 and 9.0 were detected in both pollen extracts with the rNtD-specific antiserum, ash pollen showed a higher number of basic isoforms than olive pollen. On the other hand, the main spots recognized with the rCtD-specific antiserum are present in both extracts (Fig 6A).

We performed an ELISA assay to compare the IgE-inhibition capability of ash and olive allergens from pollen extracts when purified rNtD and rCtD of Fra e 9 were coated in plates and immunostained with a pool of six Ole e 9-allergic sera (Fig 6B). In both cases a 100% inhibition was reached with the highest concentrations used. Thus β-1,3-glucanases from both extracts share IgE epitopes.

## Discussion

In this study an allergenic β-1,3-glucanase enzyme belonging to the GHF 17 family has been identified in ash (*Fraxinus excelsior*) pollen. Proteins such as xylanases, pectin methylesterases and β-glucanases are enzymes involved in carbohydrate metabolism during pollen germination and considered to play important roles in pollen-pistil interactions during plant reproduction. Some of these proteins from different pollens have been categorized as aeroallergens [38] because they cause IgE sensitization of patients with pollinosis. In the Oleaceae family, proteins related to carbohydrate metabolism, such as Ole e 9 (β-1,3-glucanase), Ole e 10 (carbohydrate-binding protein) and Ole e 11 (pectin methylesterase) [14, 27, 33] have been identified as relevant allergens for olive pollen allergic patients.

Beta-1,3-glucanases from many biological sources have been described as allergens-Hev b 2 from latex, Mus a 5 from banana and Ole e 9 from olive pollen-. Their widespread presence in plants makes them important targets for study. The clinical relevance of the ash β-1,3-glucanase could be very important if we consider that: i) *Oleaceae* family is a source with many allergens characterized as major inducers; ii) the high relevance of this pollinosis in many European countries [39, 40]; and iii) the cross-reactivity associated to many of the *Oleaceae* allergens [20, 41]. The mature Fra e 9, from the sequenced clone herein reported, renders a 432 amino acid polypeptide in length with a pI of 8.52 and shows similarity to β-1,3-glucanases. However, more isoforms are detected in the 2D-PAGE of the ash pollen extract (Fig 6). The mature enzyme with a molecular mass of 46948.3 Da belongs to the group of long glucanases composed of two functional domains, a glycosyl hydrolase catalytic N-terminal domain and a carbohydrate-binding module at the C-terminus, which belongs to the family 43 and whose first described member was another allergen from olive pollen, Ole e 10 [27]. A proline-rich segment, of 20 residues including 8 prolines, connects the two neighbouring domains. This type of enzymes could be moderately acid as Ole e 9 from olive pollen [14] or with a basic pI such as the A6 protein from *A*. *thaliana* or *Brassica* [42] and rFra e 9, and they seem to be specifically expressed in reproductive organs of higher plants, Ole e 9 [14] in pollen and A6 in anthers. The fact that Fra e 9, belongs to the same family of proteins within *Oleaceae* family could suggest a tissue-specific location also for Fra e 9. Purification of the β-1,3-glucanases directly from the pollen grain turn into a hard task when large gene families encode several isoforms of the enzyme or/and they are expressed at low levels. Both circumstances coincide in this enzyme and, although several attempts have been performed to isolate homogeneous preparations of β-1,3-glucanase from ash pollen, its low yield has required a recombinant methodology in order to have the protein available. The production of the whole Fra e 9 has been attempted using different systems and strategies, but none of them has rendered satisfactory results. The previous production of the independent domains of Ole e 9 with very good yield [20, 24] prompted us to use the same approach for Fra e 9. Although we had previously used the yeast *P*. *pastoris* [20] to get the rNtD-Ole e 9, we used *E*. *coli* for rNtD-Fra e 9. Bacteria is an appropriate host for the expression of β-1,3-glucanase enzymes because it has no endogenous enzymatic activity neither for β-1,3-glucanase nor for other enzymes that modified polymers in the plant cell wall [10]. The N-terminal domain of 36 kDa (320 amino acids) and pI 9.04 shares the characteristic (β/α)_8_ TIM-barrel fold [37, 43] and includes in the catalytic active site all the critical residues involved in the disruption of β-1,3-glucans. The ability of the rNtD-Fra e 9 to hydrolyse β-1,3-glucans as laminarin is 200-fold higher than that of Ole e 9 allergen or the A6 protein [42] in which a low or null activity, respectively, was detected. The pI differences between the NtD of Ole e 9 and Fra e 9, 5.50 versus 8.52, could be the reason of this higher activity always attributed to the basic and shorter members of this family of enzymes [22]. In fact, the activity is comparable to that of the short basic enzyme of barley [22]. The pH dependence of enzymatic activity was measured at a pH interval from 3.5 to 8.0, showing the highest activity at the range from pH 4.5 to 6.0 as most of the glucanases from GHF-17 family [7] such as banana β-1,3-glucanase with an optimum at 4.5 [44].

The network of three disulphide bonds in the CtD of Fra e 9 is the reason why the methylotrophic yeast *P*. *Pastoris* was considered as a suitable expression host for this protein in order to obtain the correct disulphide bridges network [24, 45]. In comparison with CtD-Ole e 9, the disulphide bonds and most of the aromatic amino acids-tryptophan and tyrosine residues- are also conserved in CtD-Fra e 9. These residues are involved in the stacking interactions with the sugar rings of the ligand or substrate [23]. Considering soluble polysaccharides such as laminarin, rCtD-Fra e 9 binds to this β-1,3-glucan in a gel-affinity electrophoresis. The independent folded Cys-rich domain could be implicated in the regulation of the catalytic activity of the whole enzyme and in fact this domain could bind polysaccharides bringing the biocatalytic site into close vicinity with the substrate, allowing the carbohydrate hydrolysis as it has been supported by previous reports with other enzymes containing CBM-43 modules [23].

Although patients allergic to Oleaceae species such as olive, ash, privet and lilac, have been reported, only ash and olive pollen are considered important sources of allergy. A high sequence identity is generally observed between homologous proteins from different members in *Oleaceae* family, giving rise to a strong cross-reactivity among patients allergic to these pollens. As an example, Ole e 1 and their counterparts in privet (Lig v 1), lilac (Syr v 1), and ash (Fra e 1) exhibited more than 85% of identity for their amino acid sequences [46]. However, remarkable differences attributed to protein yield or sequence similarity were detected in the *Oleaceae* β-1,3-glucanases. In fact, Fra e 9 shared higher identity with glucanases from pear (56%) than with Ole e 9 (40%). The comparison of the catalytic domain of Fra e 9 (NtD) with other short glucanases, such as those from latex, banana and tomato, rendered as well low percentages of amino acid sequence identity (31%, 33% and 34%, respectively).

Although the cross-reactivity is an important aspect to consider between the ash and olive *Oleaceae* allergens, the relevance of Fra e 9 as allergenic sensitizer is particularly important in those populations from areas where the ash pollen grains levels are high and the presence of olive tree does not interfere. In populations such as Strasbourg (France) or the north of Spain, ash pollen allergy is significant [39] and the prevalence for Fra e 9 can reach the 60% [40].

In conclusion, Fra e 9 is a new analysed β-1,3-glucanase belonging to the *Oleaceae* family. Despite of belonging to the same phylogenetic family, it exhibits sequence identity differences that could explain its higher hydrolytic capacity against laminarin than that of its counterpart in olive pollen. However, both proteins retain the ability to bind IgE from sera of allergic patients. Therefore, β-1,3-glucanases represent a heterogeneous enzyme family with intrinsic allergenic capacity.

## Supporting Information

S1 File Oligonucleotides used for Fra e 9 PCR amplification. The coding region of nucleotide sequences in capital letters and the amino acid sequence are indicated. Sites for restriction enzymes are underlined.
(DOCX)
