# Supplementary material for: An Enzymatically Active β-1,3-Glucanase from Ash Pollen with Allergenic Properties: A Particular Member in the Oleaceae Family
Source: PLoS One. 2015 Jul 15;10(7):e0133066. doi: 10.1371/journal.pone.0133066 (PMC4503641; doi:10.1371/journal.pone.0133066)
Supplement: S1 File — The coding region of nucleotide sequences in capital letters and the amino acid sequence are indicated. Sites for restriction enzymes are underlined. (DOCX) [file pone.0133066.s001.docx]

**S1 Table. Oligonucleotides used for Fra e 9 PCR amplification.** The coding region of nucleotide sequences in capital letters and the amino acid sequence are indicated. Sites for restriction enzymes are underlined.

| **Oligonucleotide** | **Nucleotide sequences** | **Strand** | **Amino acid sequences** |
| --- | --- | --- | --- |
| **Fra9.1** | 5'- TCNTTYATHGGNGTNAAYTAYGG -3' | Sense | SFIGNVNYG (29-35) |
| **Fra9.2** | 5'- NGGCCANCCNGTYTCNGCNAC -3' | Antisense | VAETGWP (262-268) |
| **Fra9.3** | 5'- GGCTATGGCGATGTGGAGATTGC -3' | Sense | GYGDVEIA (254-261) |
| **UPM** | 5'- CTAATACGACTCATAGGGCAAGCA  AGCAGTGGTAACAACGCAGAGT -3' | --- | --- |
| **Fra9.4** | 5'- ATGGAGTTTTCAAAACGACAC -3' | Sense | MEFSKRH (1-7) |
| **CtD.2** | 5'- ctgcggccgcTCAAGAGAGGAATGAGCACG -3' | Antisense | SCSFLS (463-468) |
| **NtD.1** | 5'- atacatatgATCGGAGTTAATTACGGC -3' | Sense | IGVNYG (30-35) |
| **NtD.2** | 5'- cggaattcTCAGTGGTGGTGGTGGTGG  TGGGTTCCTCGCATGATTCCAAC -3' | Antisense | VGIMRGT (343-349) |
| **CtD.1** | 5'- gtctcgagaaaagaGCAGGTGTGCCTGATCAG -3' | Sense | AGVPDQ (250-256) |
